# Draft Genome Sequence for ICMP 5702, the Type Strain of *Pectobacterium carotovorum* subsp. *carotovorum* That Causes Soft Rot Disease on Potato

**DOI:** 10.1128/genomeA.00875-15

**Published:** 2015-08-06

**Authors:** Preetinanda Panda, Ashley Lu, Karen F. Armstrong, Andrew R. Pitman

**Affiliations:** a Bio-Protection Research Centre, Lincoln University, Canterbury, New Zealand; b The New Zealand Institute for Plant & Food Research Limited, Lincoln, Canterbury, New Zealand

## Abstract

*Pectobacterium* species are economically important bacteria that cause soft rotting of potato tubers in the field and in storage. Here, we report the draft genome sequence of the type strain for *P. carotovorum* subsp. *carotovorum*, ICMP 5702 (ATCC 15713). The genome sequence of ICMP 5702 will provide an important reference for future phylogenomic and taxonomic studies of the phytopathogenic *Enterobacteriaceae*.

## GENOME ANNOUNCEMENT

Enterobacterial pathogens belonging to the species *P. carotovorum* are ubiquitous in nature and infect a broad range of crops including Brussels sprout, carrot, celery, cucumber, banana, tomato, capsicum, and potato (1–8). Isolates of *P. carotovorum* have been divided on the basis of differences in host preference and their ability to survive in different geographical environments (9). They have also been separated using DNA-DNA hybridization, numerical taxonomy, phylogenetic analysis, and serology. This has resulted in the recognition of several subspecies of *P. carotovorum*, including *P. carotovorum* subsp. *carotovorum*, *P. carotovorum* subsp. *odoriferum*, *P. carotovorum* subsp. *betavasculorum*, and *P. carotovorum* subsp. *brasiliensis* (10–12).

The nomenclature of the plant pathogenic enterobacteria has undergone a variety of taxonomic revisions and reclassifications. Genome sequencing provides an opportunity to clarify the phylogenomic and taxonomic space of the genus *Pectobacterium*, facilitating efforts to assign evolutionary relationships and identify new species. As type strains play a crucial role in such studies, providing a fixed reference point for the assignment of bacterial names and exhibiting all of the relevant phenotypic and genotypic properties cited in the original published taxonomic circumscriptions, the sequencing of the genomes for type strains is imperative. Here, we report the draft genome sequence of ICMP 5702, the type strain for *P. carotovorum* subsp. *carotovorum*.

The draft genome of ICMP 5702 was sequenced using a HiSeq 2000 system to generate 100-bp Illumina paired-end reads (Axeq Technologies, USA). The quality of the sequence reads was checked, and low-quality reads (score of <Q30) were trimmed using FastQC (Baraham Bioinformatics, United Kingdom). A *de novo* assembly was then performed with the edited sequence reads using SOAP *de novo* v2.04 (13), resulting in a draft genome sequence for ICMP 5702 with a coverage of ~600×. The draft genome is 4,774,457 bp in size and comprises 46 contigs with a G+C content estimated to be 51.9%.

The draft genome sequence of ICMP 5702 was annotated using the PGAAP annotation pipeline (http://www.ncbi.nlm.nih.gov/genome/annotation_prok/). Annotation revealed the presence of 72 tRNAs and 4,109 coding sequences, a number encoding pathogenicity- or virulence-related factors such as plant cell wall degrading enzymes, flagella-based motility, cell membrane structures such as lipopolysaccharides and secretion systems. The identification of these factors provides insight into the mechanisms used by *P. carotovorum* subsp. *carotovorum* to invade potato tubers. As the type strain for this species/subspecies, the draft genome of ICMP 5702 also provides an important resource for future phylogenomic and taxonomic studies of phytopathogenic enterobacterial pathogens.

### Nucleotide sequence accession numbers.

The draft genome sequence of *P. carotovorum* subsp. *carotovorum* ICMP 5702 has been deposited in the GenBank database under the accession no. AODT00000000. The version described in this paper is the first version, AODT01000000.
